# Medical staff opposition to a deep and continuous palliative sedation request under Claeys-Leonetti law

**DOI:** 10.1186/s12904-018-0384-3

**Published:** 2019-01-08

**Authors:** Claire Vitale, Alexandre de Nonneville, Marie Fichaux, Sebastien Salas

**Affiliations:** 1 Department of Oncology and palliative care, Timone Hospital, Marseille, France; 2 Paul Valéry University, Montpellier, France; 3 Aix-Marseille University, Marseille, France; 4 Service Oncologie médicale et Soins Palliatifs, C.H.U. Timone, 264 rue Saint Pierre, 13005 Marseille, France; 5 Université Paul Valéry, Route de Mende, 34090 Montpellier, France; 6 Faculté de Médecine - Timone 27, Aix-Marseille Université, Boulevard Jean Moulin, 13385 Marseille, France

**Keywords:** Sedation, End-of-life, Palliative care, Claeys-Leonetti, Medical staff opposition

## Abstract

**Background:**

For the year 2018, the French government plans a revision of bioethics laws, including certainly the recent Claeys-Leonetti law introducing the right to deep and continuous sedation at the request of palliative patients and prohibiting euthanasia for end-of-life patients. Because there is no published data on medical staff opposition to a deep and continuous palliative sedation request under Claeys-Leonetti law, we believe this report may give insight into physicians’ decision making, into the role of criteria for prudent practice, and thus contribute to the bioethical debate.

**Case presentation:**

We report a 70-year-old patient with squamous cell carcinoma of the hypopharyngeal region, who categorically refused any treatment since one year and asked for deep and continuous palliative sedation until death after attempting suicide. The patient’s request was examined and denied by palliative multidisciplinary board, in accordance with by the French Oncology Coordination Centre guidelines. This situation did not fulfil the criteria requested by Claeys-Leonetti law.

**Conclusions:**

As highlighted by the present case-report, patient’s expectation regarding palliative sedation can be ambivalent with properly so called euthanasia or assisted suicide. This ambivalent perception was part of the controversy surrounding the parliamentary debate, which is still relevant. This case report supports that deep and continuous sedation under Claeys-Leonetti law need to meet specific criteria defined by the law and documented in the medical files as a safeguard against inappropriate practice. In fact, one of the shortcomings of the current arrangements of Claeys-Leonetti law is a lack of objective medical-based criteria. So it is necessary that scientific peer-reviews papers be published quickly in order to deepen the bioethical debate on the end of life.

## Background

Since February 2, 2016, the French Government proclaimed a law called the Claeys-Leonetti law [1], after the re-examination of questions related to accompaniment of patients at the end of life and euthanasia. Indeed, the first law concerning the rights of patients at the end of life and called Leonetti law [2] was unable to respond to public debate on the issue of euthanasia and appeared limited concerning patient’s rights. Indeed, if the Leonetti law allowed terminal sedation, it did not allow deep sedation and continues until death at the patient’s request. Thus, terminal sedation was already authorized by the Leonetti law when patient experiences acute complications with immediate vital risk, situations that may be complicated by an immediate vital risk (cataclysmic haemorrhages and asphyxia respiratory distress), refractory symptoms or when the patient is no longer able to express his will and as a refusal of unreasonable obstinacy the doctor decides to stop a maintenance treatment alive (respirator). According to the European Association for Palliative Care (EAPC) “Palliative sedation was defined in analogous ways in all guidelines, that is, as an intervention instituted solely for the purpose of refractory symptom control. It could be light (or superficial) or deep (patient is asleep and unresponsive). This could be intermittent and temporary, or continuous until death” [3].

The Claeys-Leonetti law, that always forbade euthanasia or slaw-euthanasia (described as occurring “when clinicians sedate patients approaching the end of life with the primary goal of hastening the patient’s death” [4]), maintains terminal sedation, but also increased patients’ autonomy by strengthening the value of advance directives and extending the spectrum of unreasonable obstinacy to the sustainment of vital treatments [5]. Claeys-Leonetti law improved end-of-life conditions by establishing, under certain conditions, the right to deep and continuous sedation at the request of palliative patients, consisting of sedative treatment, and analgesic treatment if needed, leading to a profound and continuous change of vigilance to death, associated with the cessation of all life-sustaining treatments including artificial nutrition and hydration [6].

The set-up of this law forces doctors, after discussing the case in palliative multidisciplinary meeting and in accordance with the French Oncology Coordination Center recommendations to implement it, at the patient’s request, under conditions. The establishment of deep and continuous sedation at patient’s request is permitted by law in only two situations. First, deep and continuous sedation at patient’s request is indicate when a seriously ill and incurable patient with a short-term prognosis (this term is not made concrete in the law) experiences refractory symptoms. Secondly, when incurable patient with a short-term pathology prognosis take the decision to stop a treatment that could result in a short term life-threatening and/or potential unbearable suffering (artificial hydration is considering as a life-sustaining treatment only since the application of Claeys-Leonetti law).

Since the approval of the law, we have faced several requests for sedation [6]. They were performed after validation by our palliative multidisciplinary meeting, in accordance with by the French Oncology Coordination Centre recommendations. Recently, we refused a sedation request to a palliative care patient. Because there is no published data on medical staff opposition to a deep and continuous palliative sedation request under Claeys-Leonetti law, we believe this report may give insight into physicians’ decision making and into the role of criteria for prudent practice.

## Case presentation

A 70-year-old man with antecedent of follicular lymphoma in complete remission presented at the Timone University Hospital (Marseille, France) in 2016 for a squamous cell carcinoma of the hypopharyngeal region. The patient categorically refused any treatment, including preservative surgery, radiotherapy, chemotherapy or supportive care.

One year later, he was addressed to our palliative care unit by the hand-surgery department after attempting suicide. The patient explained his action by the fear of suffering. No depressive state was diagnosed by our psychiatrists. Despite persistence fear of suffering, the patient rejected the idea of suicide because of his family, but still wanted to die and asks for assistance. Information on Claeys-Leonetti law was given, especially on assisted-suicide banishment and on the possibility to relieve suffering with adapted treatments.

One week after discharge, the patient was readmitted to our department for dyspnea and anxiety. Symptoms were managed by appropriate treatments (oxygen and low dose of midazolam in an anxiolytic purpose). Despite stabilisation, the patient was afraid of dying suffocated and asked for deep and continuous palliative sedation until death. Apart from the fear he expresses, the patient has no symptoms of anxiety, depression or pain after the introduction of appropriate treatments. On the other hand, he clearly states that he refuses to live again knowing that his death is approaching and that he is apprehensive of suffering. He says he wants to rush his death. For us, this is a request for assisted-suicide (active help from a third party for the administration of a lethal product) or euthanasia (act of a third party which intentionally provokes the death of another to put an end to his sufferings), rather than a real demand for deep and continuous sedation. It seems important to note that patient’s requests for deep and continuous sedation until death are not registered officially. The law does not even impose a written request. Thus, the request is most often made orally in the presence of several doctors and clinicians.

In order to try to objectify this request and therefore our answer, the patient’s request was examined and denied by palliative multidisciplinary board, in accordance with by the French Oncology Coordination Centre guidelines. This situation did not fulfil the criteria requested by Claeys-Leonetti law. Indeed, prognosis appeared not short term committed (no visible clinical progression of the disease, which commits for sure the short-term vital prognosis), symptoms were managed with appropriate treatments and no life-sustaining treatment arrest could lead to potential unbearable sufferings. Regarding the short-term criterion of life-threatening prognosis, the patient was offered to have a Computed Tomography (CT) scan to measure the progression of the disease. Indeed, no imaging had been performed for one year (time of diagnosis of recurrence). The patient refuses this proposal. The request for deep and continuous sedation was reiterated several times by the patient, who was still refusing any investigations to define the progression of his cancer and wanted parenteral hydration to be maintained. Daily, he questioned each caregiver about the rationale for the refusal of his request. How can the medical staff be sure that his prognosis is not short-term compromise? Why his psychological distress could not be considered as refractory? One week after refusing further investigation, the patient finally agrees to undergo a CT scan. Three days after the exam he dies peacefully, according to our team (no specific questionnaires or objective elements to judge the quality of death exists), of a not predictable respiratory distress certainly linked to the evolution of his cancer of the hypopharyngeal region without introduction of deep and continuous sedation, but with introduction of midazolam for anxiety. Opiates were not introduced because the patient was saying not being painful. The CT scan results, unknown at the time of death, reveal nothing conclusive (pulmonary metastases, but no lymph node involvement) and would have required additional analyzes.

## Discussion and conclusions

The Claeys-Leonetti law created the right to deep and continuous sedation. In a recent publication [7], we pointed that this right was eagerly awaited by palliative care patients. An opinion survey conducted among patients treated in different palliative care institutions showed that up to 83% palliative care patients were in favor of the right to deep and continuous sedation. Nevertheless, 53% of patients reported opposition to legalized euthanasia. Despite these different opinions between palliative sedation and euthanasia, patient’s expectation regarding palliative sedation can be ambivalent with properly so called euthanasia or assisted suicide, as highlighted by the present case-report. This ambivalent perception was part of the controversy surrounding the parliamentary debate [8]. Some regard the practice of palliative sedation as proper medical care, whereas others see continuous sedation as slow, disguised, and socially acceptable form of euthanasia. Moreover, our clinical case raises the question of intention in deep and continuous sedation until death at the patient’s request. This problem of intention, already put into work for sedation, has been partly resolved by the principle of double effect. Here he is exacerbated by the fact that the patient is asking for sedation.

This case report supports that deep and continuous sedation under Claeys-Leonetti law need to meet specific criteria defined by the law. Criteria for application of palliative sedation should be clearly identified and documented in the medical files as a safeguard against inappropriate practice. Prudent practice involve standardized decision-making model for medical team guidance. Implementation of a medical ethics decision-making model is quite similar to that of medical guidelines [9].

One of the shortcomings of the current arrangements of Claeys-Leonetti law is a lack of objective medical-based criteria. It appears through our patient case that the application of Claeys-Leonetti law challenge two main areas of Western medical system: diagnosis and prognosis. Diagnosis of psychic suffering in an objective ways could be difficult. Qualified it as “refractory” to treatment is even more challenging. The difficulty of evaluating a “refractory symptom”, accentuated when it comes to so-called psychological or existential symptoms, is put forward by the EAPC.

After defining the refractory symptom as precisely as possible, the EAPC framework discusses the relevance of considering the physicians or other clinicians being in the best position to determine whether a symptom is refractory. “However, it is not clear why clinicians are in the best position to determine whether or not a symptom is refractory, among other things because it is questionable whether clinicians are in a better position than the patient to determine whether or not an intervention provides “adequate relief”” (p. 2) [10]. Finally, The EACP framework recognizes the “subjectivity of refractoriness”, but “existential intolerable suffering in the end of life would, accordingly, be an insufficient indication for palliative sedation” (p. 3) [11]. This problem is not solved regarding sedation. Thus, it seems necessary to clarify the law in this place, but also to define more clearly in framework what is meant by psychic or existential suffering at the end of life, to objectify the criteria.

In another hand, the law allows palliative sedation at the request of a patient when short term poor prognosis is engaged. This point raises the question of an exact definition of “short term poor prognosis” which is not clearly specified by the law. An analysis of international guidelines and position on palliative sedation showed that the concept of short-term prognosis was unclear [12], especially it has been demonstrated that treating physicians appear to overestimate the duration of life’s patient at the end of the care of cancer patients [13]. In order to reduce this lack of objectivity, it would be interesting to develop more prediction software in purpose to specify the short-term prognosis (as maching learning for example), but also strengthen palliative multidisciplinary meetings in the presence of physicians or clinicians who do not know the patient. Considerable variation was observed in physician-reported performance and decision-making [14], highlighting the importance of providing clearer guidance on the specific needs of the context in which continuous deep sedation until death is to be performed. Moreover, there are major gaps in end-of-life laws knowledge among medical specialists involved in end-of-life [15]. Ongoing education is needed to ensure that specialists have up-to-date knowledge of the law to avoid compromising patient care or putting medical practitioners at legal risk.

Finally, through our case report, and because the patient’s request was to hastened death, we question the impact of the ambivalent perception of deep and continuous sedation at patient’s request on the medical decision. In fact, it appears that the patient’s request to provoke or hasten his death could influence our decision to refuse or accept deep and continuous sedation, because it questions the grounds of the intention of physicians and clinicians in setting up sedation. Often, the limit between relieving and giving death is tenuous. Thus, for the sake of practices’ homogenization and to prevent potential drifts due to the lack of precise criteria and objectives of the Claeys-Leonetti law, we could recommend, in addition to some degree of consensus among practitioners in palliative multidisciplinary meetings, an oversight via reporting requests and case of deep and continuous sedation until death to the central authorities, at least and for the beginning at regional hospitals.

## Abbreviations

CT: Computed Tomography
EAPC: European Association for Palliative Care
